# An Overview on Quantum Dot-based Nanocomposites for Electrochemical Sensing on Pharmaceutical Assay

**DOI:** 10.22037/ijpr.2021.115279.15291

**Published:** 2021

**Authors:** Leyla Karadurmus, Goksu Ozcelikay, Sena Vural, Sibel A. Ozkan

**Affiliations:** a *Department of Analytical Chemistry, Faculty of Pharmacy, Ankara University, Ankara, Turkey. *; b *Department of Analytical Chemistry, Faculty of Pharmacy, Adıyaman University, Adıyaman, Turkey.*

**Keywords:** Quantum dots, Electroanalytical methods, Voltammetry, Drug analysis

## Abstract

Quantum dots (QDs) are one of the first nanotechnological materials to be integrated with sensor technologies and have been widely anticipated to eventually find application chances in several commercial pharmaceutical and clinical products. They are one of the most important developments in the rapidly growing world of material science technology. The excellent properties of QDs may allow the design of simple, precise, and inexpensive electrochemical methods for the detection of pharmaceuticals. Electrochemical techniques offer accuracy, high sensitivity, low cost, simplicity, ease of preparation of the samples in a very short time, and speed of analysis. The most commonly used voltammetric techniques are differential pulse voltammetry, cyclic voltammetry, square wave voltammetry, and stripping voltammetry. The purpose of this review is to show and communicate the advantages and uses of QD applications used in drug analysis. Besides, the present application methods of QDs to the pharmaceutical analysis and their related parameters were summarized between 2012 and 2021 years and summarized as a table.

## Introduction

Nanotechnology is a very popular topic for the scientific world today. In recent years, QDs have received great attention in the detection of pharmaceuticals in different sample matrices, *in-vitro* bio-imaging, and *in-vivo *applications. QDs are widely applied to detect many analytes such as ions, pharmaceuticals, small molecules, and biological macromolecules (1, 2).

In the voltammetric technique, a quantity concerning an analyte is obtained by measuring the current produced by the change of potential. The particular chemical is related to the peak current and the concentration of the corresponding species is related to the density of the peak current. The voltammogram, which is a plot of potential versus current, shows the behavior of the chemical reaction. The main advantages of voltammetry are the ability to simultaneously detect multiple analytes with different peak potentials and the low noise of the measurements leading to very high sensitivity. Voltammetric methods include cyclic voltammetry, differential pulse voltammetry, square wave voltammetry, linear sweep voltammetry, and stripping voltammetry. Cyclic voltammetry is one of the most used methods to measure electrochemical reaction rates and redox potential (3–6).

This review presents the applications of various electrochemical modes on QDs modified electrodes, modiﬁcation style, and their related parameters in the analysis of drugs and pharmaceutically active compounds from their dosage forms and biological media. Examples of different types of applications have been reported and as with all other aforementioned techniques. Moreover, it should be kept in mind that the electrochemical techniques have not only advantages but also limitations. Also, in this review, detailed information about quantum point nanomaterials and new applications on pharmaceutical analysis using quantum point-based nanosensors, advantages and disadvantages of quantum point nanosensors, and future perspectives will be given.

**Quantum dots**

**Figure 1:**
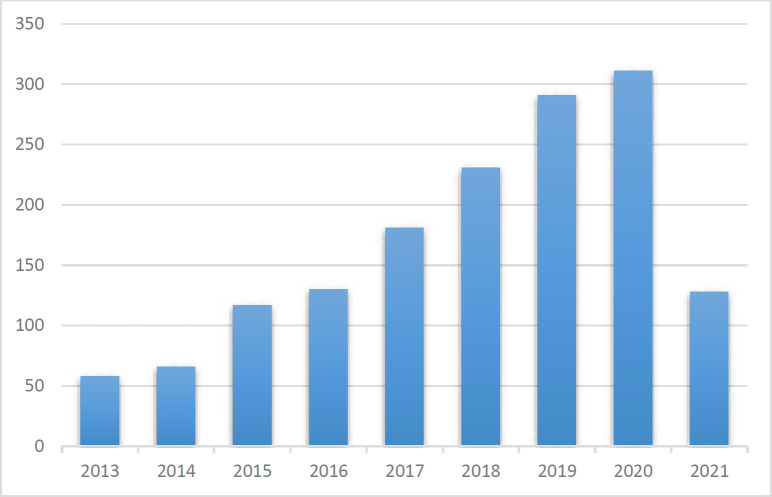
Statistics of the number of publications per year related to quantum dot-based nanocomposites for electrochemical sensing

The detection of pharmaceuticals is an important aspect of therapy safety. A range of detection techniques and novel materials have been developed to achieve rapid, sensitive, and precise monitoring of certain analytes. Nanomaterials with unique electronic, optical, mechanical, and thermal properties have been accepted as one of the most up-and-coming materials for opening new gates in the development of new analytical methods for the analysis of drugs. Nanomaterials indicate novel properties that present great opportunities for the improvement of new analytical methods for the analysis of drugs. In recent years, researchers have shown a great interest in the production of nanoparticles such as quantum dots, nanowires, nanotubes, nanorods, or nanofilms. Statistics of the annual number of publications on quantum dot-based nanocomposites for electrochemical detection in the last eight years are given in Figure 1. The excellent electrical and optical features of nanomaterials, such as quantum dots, carbon nanotubes, gold nanoparticles, nanorods, graphenes, and nanopores, are closely related to their sizes (7–11). Quantum dots (QDs) are nanoscale semiconductor materials, such as cadmium selenide (CdSe). Today, the most frequently generated quantum dots due to their optical and electrical properties are CdSe, InAs, CdS, GaN, InGeAS, CdTe, PbS, PbSe, ZnS. In quantum dots, size is a controllable parameter, which, when combined with the effect of quantum restriction, creates quantum dots with extraordinary optical and electrical properties. Quantum dots (QDs), usually semiconductor nanocrystals of 2-6 nm, are one of several nanomaterials that significantly impact research in many areas, such as chemistry and biology (12–15). 

Researchers have employed QDs as labeling materials for biosensors. An extensive review of the improvement of assays and nanosensors using QDs as components is presented. QDs are of great interest in the development of optical probes for cellular, tissue, or whole-body imaging and biological detection (16). As a unique nanomaterial, QD-based sensors offer high sensitivity and selectivity in detecting certain analytes in the chemical and biochemical sciences. Integrated with QDs, electrochemical sensors have led to the improvement of highly selective and efficient analytical techniques. QDs can significantly increase the density of the electrochemical signal in the electrochemical detection system and supply sharp and well-resolved voltammetry signals. In sensor technology, QD-based sensors are very suitable for creating highly selective, rapid, and precise tools for the detection of specific analytes (6, 17–22)

**Electroanalytical Methods in Drug Analysis**

Stability testing, quality control, and analysis of the development of a new pharmaceutical product have led to the continuous development of analytical methods (23, 24). There are many suitable methods for determining the content of the drug substance or active ingredients in pharmaceutical formulations and biological samples (25). Various methods such as chromatography, ultraviolet spectrometry, nuclear magnetic relaxation spectroscopy, capillary electrophoresis, and high-performance liquid chromatography have been used. However, these methods require expensive instruments, complex procedures, and specific sample pre-treatments (26). 

Electroanalytical methods can be divided into various sub-divisions based on applying either potential or current and/or measuring potential, current, impedance, etc. In electroanalytical techniques, voltammetry is the leading method. Voltammetric techniques are also divided into subgroups such as cyclic voltammetry (CV), differential pulse voltammetry (DPV), and stripping methods. Amperometry is the other electroanalytical technique in which mostly used for the current measurements after the application of a constant potential. Electrochemical impedance spectroscopy (EIS) is one of the most comprehensive methods for the characterization of electrochemical systems with measuring resistive and capacitive properties. Electrochemical methods have attracted great attention due to their advantages in the field of drug analysis. These advantages include a wide range of linear concentrations, inexpensive, fast analysis times, simultaneous determination of several analytes, and the ability to measure small currents. It allows measurements to be performed with very small sample volumes in the microliter range (2, 27 and 28). For these reasons, electrochemistry is an appropriate method of analysis for the analysis of drugs. Besides, electrochemical methods can be used for *in-vivo *analysis of drugs. Voltammetry is the most widely used electroanalytical method. Voltammetry has a growing field of application due to its advantages in drug analysis. The voltammetric methods take advantage of explaining the oxidation and reduction effects of drug substances and pharmacological action mechanisms (5, 29). Commonly used voltammetric techniques are differential pulse, cyclic, square wave, and stripping voltammetry. Cyclic voltammetry (CV) is used to provide significant information about the oxidation/reduction mechanism of the drug active compounds, and techniques such as different pulses, square wave, and stripping voltammetry are used to determine the small volume of the drug(30). The performance of voltammetric methods depends to a large extent on the material of the working electrode. The voltammetric method uses a wide variety of solid electrodes, such as various carbon electrodes, noble metal electrodes, and modified electrodes (31). To increase selectivity on the electrode surface, it is necessary to change the surface quality, briefly change the electrode surface. Furthermore, it is possible to create a surface with an elongated and stable chemical structure giving reproducible results, and as the sensitivity and selectivity increase, the working potential range expands.

To summarize the numerous recent applications of voltammetric methods for the analysis of drugs, we listed the information on the electrode, supporting electrolyte, voltammetric mode, and detection limit in Table 1.

**Recent applications on pharmaceutical analyses using quantum dots based nanosensors**

A sensor is a device that can transform the physical, biological, or chemical property of a system into an analytically measurable, processable, and useful signal by a transducer. If the sensor includes a nanoscaled interaction, it is described as a nanosensor. Quantum dots have attracted much interest from researchers because of their unique optical, electrical, thermal, and catalytic properties and have been used in the construction of various electrochemical sensors. This review describes a few examples to illustrate the administration of electrochemical techniques for pharmaceutical and drug analysis. Special attention has been shown to voltammetric analyzes using quantum dots modified electrodes. Several articles are published every year related to the voltammetric analysis with quantum dots modified electrodes of pharmaceuticals. The publications related to the modification of quantum dots can be shown as follows. 

Tang *et al.* have constructed an electrochemical sensor using a glassy carbon electrode (GCE) modified with graphene quantum dots (GQDs) for the determination of hydroquinone and catechol in 2018 (32). This sensor was designed by the electrodeposition method and characterized by electrochemical impedance spectra. The proposed GQD’s sensor revealed a very good sensitivity, reproducibility, and reliability in the electrochemical measurement, obtaining the detection limit down to 0.08 μM in the range from 0.5 μM to 100 μM. Simultaneous detection of HQ and CC with GQD/GC electrode was performed in river water samples with good recovery. In this study, the advantages of the proposed sensor, such as excellent electrocatalytic and conductivity properties and high precision, reliability, and reproducibility in electrochemical measurement, were utilized for HQ and CC.

A novel, highly sensitive, and selective CdS quantum dots (QDs) modified carbon paste electrode (CPE) was developed by Pasandideh-Nadamani and co-workers in 2016 (33). They synthesized quite stable CdS QDs, which are characterized by X-ray diffraction (XRD) and transmission electron microscopy (TEM) techniques. CdS QDs were obtained in an *in-situ* technique using a thiosulfate precursor. The electrochemical determination of p-aminophenol (PAP) and acetaminophen (Ac) was investigated without any separation steps in the mixture. 

Algarra and co-workers have constructed carbon quantum dots (CQDs) modified glassy carbon electrode (GCE) electrochemical determination of dopamine and acid uric (34). CQDs were obtained from graphite by the Hummers method and were characterized with various methods such as TEM microscope, XPS, Raman, solid-state NMR, and FTIR-ATR spectroscopies. The electrochemical determination of both compounds showed a significant enhancement in the peak current in the CQDs-GCE as compared to the bare glassy carbon electrode. By Linear Sweep Voltammetry (LSV), the proposed sensor exhibited high sensitivity. The lower limits of detection were found to be 1.3 μM and 2.7 μM for uric acid and dopamine, respectively.

By Wong and co-workers, an electrochemical method employing a cadmium telluride quantum dots (CdTe) in Printex 6L Carbon (P6LC) and within a poly(3,4-ethylene dioxythiophene) polystyrene sulfonate (PEDOT:PSS) film modified glassy carbon electrode (QDs-P6LC-PEDOT:PSS/GCE) was developed for the detection of amoxicillin (35). The morphological structures of the nanostructured material were characterized using transmission electron microscopy, X-ray diffraction, and confocal microscopy. Square-wave voltammetry (SWV) was employed to investigate the electrochemical behavior of amoxicillin. Under the optimum conditions, the obtained sensor exhibited good sensitivity, high selectivity, and stability. No significant interference was noticed from drugs and potential biological interferences such as paracetamol, ascorbic acid, uric acid, and caffeine. The proposed sensor could be used for simultaneous determination of amoxicillin in tablets, urine, and milk samples.

An electrochemical sensor has been developed for the simultaneous detection of methyldopa (MET) in tablet, urine, and human serum samples using a molding of an aliquot of thioglycolic acid capped CdSe@Ag2Se on a glassy carbon electrode by Asadpour-Zeynali and Mollarasouli (36). CdSe@Ag2Se was characterized by X-ray diffraction (XRD), scanning electron microscopy (SEM), FT-IR spectroscopy, photoluminescence spectroscopy, cyclic voltammetry, and UV–vis techniques. Differential pulse voltammetry (DPV) was used to examine the electrochemical determination of MET. Under the optimum conditions (pH 2.0), the linear methyldopa range and limit of detection are 0.09 to 60 µmol L^−1^and 0.04 µmol L^−1^, respectively. 

**Advantages and disadvantages of Quantum dots nanosensors**

**Figure 2:**
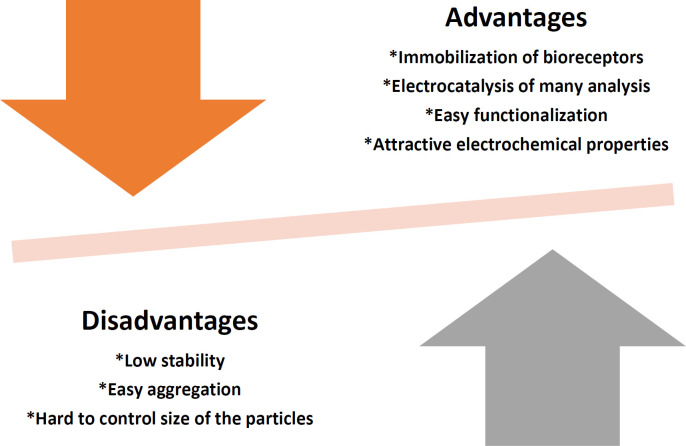
Disadvantages and advantages of quantum dots in an electrochemical sensor

Nanomaterials are ideal materials for creating sensors. In quantum dots, size is a controllable parameter, and when this property is combined with the “quantum limitation” effect, quantum dots have extraordinary optical and electrical properties. Because the size of quantum dots changes with the effect of quantum restriction, the color of their luminescence also changes. Quantum dots can be used as fluorescent probes for medical diagnosis and imaging. However, heavy metals such as CdSe, CdTe, and CdS tend to degrade under physiological conditions, and ion release is toxic (12, 37). The disadvantages and advantages of quantum dots in an electrochemical sensor are given in Figure 2.

**Table 1:** Selected quantum dots based electrochemical studies for different active compounds

Active Compound	Method	Transducer	Linear Range	LOD/LOQ	Application	Reference
Catecholamine	CV	GQD/Lac/GCE	1–120 μM	83 nM /126 nM	Pharmaceutical samples	(38)
Curcumin	DPV	CQD/GCE	0.4-200 μM	0.1 μM	Turmeric powder	(39)
Metanil yellow			0.06- 50 μM	0.03 μM		
Metobromuron	DPV	MIP/Au NPs@NCDS @Ag NPs/GCE	1 pM–2 nM	0.2 pM	Wastewater samples	(40)
Oxalic acid	Amperometric	NH2-GQD/GO/GCE	0.5-2 mM 2-55mM	50 μM	Urine samples	(41)
Metronidazole	DPV	CuCo2O4/N-CNTs/MIP/GCE	0.005–0.1 μM 0.1–100 μM	0.48 nM	Pharmaceutical samples Human serum sample Human urine sample	(42)
Caffeic acid	DPV	N-CQD/HP-Cu2O/MWCNT/GCE	0.05–43 μM	0.004 μM	Red wine sample	(43)
Quercetin	DPV	NH2- GQD/Au-β-CD/GCE	1-210 nM	285 pM	Honey Tea Honeysuckle Human serum	(44)
Sofosbuvir	DPV	MIP/AuNPs/ N,S@GQD/PGE	1–400 nM	0.36 nM	Human serum sample Pharmaceutical samples	(45)
Vitamin B2	DPV	PGBHA-NH2-GQD/MnO2 NCs/GCE	0.1 to 100 μM	0.04 μM	Real sample	(46)
Dopamine				0.05 μM		
Flutamide	DPV	N-CQD@Co3O4/ MWCNT/GCE	0.05-590 μM	0.0169 μM	Human urine sample	(47)
Nitrofurantoin			0.05-1220 μM	0.044 μM		
Oxaliplatin	DPV	CQDs@HBNNS/UiO-66-NH2 /MIP/GCE	1-250 nM	0.37 nM	Human serum sample Human urine sample	(48)
Daunorubicin	DPV	CQD/PGE	0.1 -0.5 μM	37 nM	5 mM phosphate buffer solution (pH 7.40)	(49)
Dopamine	DPV	MIP/Au/N-GOQDs/NiS2/BC/GCE	0.05–40.0 µM	0.0028	Pharmaceutical samples Human serum sample Human urine sample	(50)
Chlorpromazine			0.005–2.0 µM	0.00025		
Dobutamine	DPV	N-GQDs/NiMnO3/CPE	0.08–40.0 μM	0.02 μM	Human serum sample	(51)
p-aminophenol Acetaminophen	CV	CdS/CPE	100-1400μM /200-1200 μM	2 μM/ 10 μM	-	(33)
Cocaine	DPV	AgNPs-Apt/CdTe QDs/GCE	0.05–6000 nM	0.005 nM	Human serum sample	(52)
Doxorubicin hydrochloride	DPV	GQD/GCE	0.018–3.60 μM	0.016μM	Human serum sample	(53)
Hydroxylated polychlorobiphenyls	SWV	Tyr-ZnO QDs/GO/GCE	2.8–27.65μM	0.15 μM	Phosphate buffer solution	(54)
Methyldopa	DPV	TGA-CdSe@Ag2Se QDs/GCE	0.09- 60 μM	0.04 μM	Pharmaceutical samples Human serum sample Human urine sample	(36)
Olanzapine	CV	BMBPBP/CdS-QDs/MWCNTs/Au electrode	0.02–100μM	0.006μM	Pharmaceutical samples Human serum sample Human urine sample	(55)
Clopidogrel	AdSDPV	fMWCNT/CdSe QDs/GCE	2–40 μM 2.5 -15 μM	0.076 μM 0.30 μM	phosphate buffer solution (pH 2.14) Human serum sample	(56)
Lamivudine Tenofivir disoproxil fumarate	DPV/ EIS	Ni-CoS/ GQDs/GCE	-	56.18 μg/mL / 56.13 μg/mL	0.1 M phosphate buffer solution (pH 8)	(57)
Dopamine Tyrosine	DPV	GQD/SPE	0.1–1000 μM 1–900.0 μM	0.05 μM 0.5 μM	Pharmaceutical samples Human urine sample	(58)
Acetaminophen Ascorbic acid	DPV	GQD/GCE	5–80 μM 25–1350 μM		Pharmaceutical samples	(59)
L-DOPA	DPV	Fe3O4@GODs/ fMWCNTs/GCE	3-400 μM	14.3 nM	Sunflower seed, Sesame seed, Pumpkin seed	(60)
Doxorubicin	DPV	GQD-GCE	0.018–3.6 μM	0.016 μM	Human serum sample	(61)
Cholesterol	DPV	β-CD@N-GQD/GCE	0.5–100 μM	0.08 μM	Human serum sample	(62)
Vitamin C	CV	Fe3O4@GQDs/GCE	0.1-9 μM	-	0.1 M pH 7.4 PBS	(63)
Nitrite	SWV	MWCNT-Chit/CdTe QD-CTAB/GCE	1–100 μM 100-600 μM	0.30 μM	Pickled vegetable	(64)
Levofloxacin	DPV	PoAP/GQD/GCE	0.05 to 100 μM	10 nM	Milk samples	(65)
Lidocaine	DPV	Cd1-xMgxTe/QD-GO/CPE	5.08 – 14.4 μM	1.1 μM	Human urine sample	(66)
		Cd1-xMgxTe/QD-rGO/CPE	2.55 -14.4 μM	95 nM		
Epinephrine		Cd1-xMgxTe/QD-GO/CPE	0.43-1.49 μM	0.41 nM		
		Cd1-xMgxTe/QD-rGO/CPE	0.109- 1.49 μM	9.2 nM		
Folic acid	CV	nSe@ZnS/electrode	12-96 nM	0 nM	Pharmaceutical samples	(67)
Acetaminophen	DPV	GA@O-CQDs/GCE	0.001–10 μM	0.38 nM	Pharmaceutical samples	(68)
Clozapine	DPV/CV	NiO/GQD/GCE	3.0–1000 nM	0.55 nM	Human serum sample Pharmaceutical samples	(69)
Nevirapine	DPV	Pd@rGO/ MoS2 QDs GCE	0.1–80 μM	0.05 μM	Human serum solution	(70)
Doxorubicin hydrochloride	DPV	GQDs/Poly (TA/β-CD) /Au electrode	0.086 μM to 3.45 μM	0.012 μM	Human serum solution	(71)
Rilpivirine	DPV	CQD/MWCNT/AgNPs/GCE	1-7 nM	0.03 nM	Human serum samples Human urine sample	(72)
Irinotecan	DPV/CV	GQDs-PANI/ZnO/ GCE	0.1 - 25.μM	0.011 μM	Aqueous Solution Injection Solution Human Serum samples Human Urine Sample	(73)
5-Fluorouracil			0.1 – 50 μM	0.023 μM		
Donepezil HCl	CV	SBT/ N-CNDs/ CoNPs /PGE	1.5 nM-400 μM	0.5 nM	Pharmaceutical samples Rabbit serum solution	(74)
Zolpidem	DPV	GQDs/DMCCE	0.1–1 μM 1-10 μM	0.061μM	Pharmaceutical samples	(75)
Norfloxacin	SWAdASV	CdTe QDs/CB/ Chit/EPH/GCE	0.2-7.4 μM	6.6 nM	Pharmaceutical samples Human serum sample Human urine sample	(76)
Sotalol	DPV	MIP/AuNPs/GQD/ SPCE	0.1–250 μM	0.035 μM	Pharmaceutical samples Human serum sample	(77)
Chloroquine	CV	rGO@WS2/GCE	0.5 - 82 μM	0.04 μM	Pharmaceutical samples Human serum sample	(78)
	DPV		0.5 - 82 μM	0.04 μM		
Uric acid	DPV, CV	CdSeQD/HF-PGE	0.297-2.970 mM	0.0833 μM	Pharmaceutical samples Human serum sample Human urine sample	(79)
Creatinine			0.442-8.840 mM	0.229 μM		
6-Mercaptopurine	DPV	MIP/sol-gel/ZnO@GQDs/PGE	0.01-50.0 μM 50.0-700.0 μM	5.72nM	Pharmaceutical samples Human serum sample Human urine sample	(80)
Kaempferol	SWV	PVP/CdS QDs/CPE	0.06-2 μM 5–25 μM	0.06 μM	Pharmaceutical samples	(81)
Metronidazole	DPV	GQDs-MIPs/GNPs/GCE	0.005–0.75 μM 0.75-10 μM	0.52 nM	Human serum sample	(82)
Vitamin C	SWV	GQD/β-CD/GCE	0.01–170 μM	0.49 μM	Human serum sample	(83)
Dopamine	SWV	QDMCPE	75 nM–0.6 μM	21 nM	Pharmaceutical samples Human serum sample Human urine sample	(84)
Uric Acid			7.5 μM –1.4 mM			
Dextromethorphan	DPV	PDDA/MWCNT/CQD/PGE	2-600 μM	0.19 μM	Pharmaceutical samples Human serum sample Human urine sample	(85)
Malachite green	DPV	(GQDs/AuNp)n/GCE	0.4 - 10 μM	0.1 μM	Fish samples	(86)
L-tyrosine	DPV	β-CD/GQD/GCE	0.1 -1.5 μM	100 nM	-	(87)
Acetaminophen	DPV	Fe3O4@SiO2-PDDA-CNT/GCE	10-110 μM	39 nM	-	(88)
Isoproterenol	DPV	GQDs/SPE	1.0 - 900.0 μM	0.6 μM	Human urine sample	(89)
Methyldopa	SWV	GQDs-IL/CPE	0.04-750 μM	0.01 μM	Pharmaceutical samples Human serum sample	(90)
Theophylline	DPV	GQD/SPE	1.0– 700.0 μM	0.2 μM	Theophylline oral solution Urine	(91)
Topotecan	DPV	ds-DNA /GQD/IL/CPE	0.35–100.0 μM	0.1 μM	Human serum sample Human urine sample	(92)
Imidacloprid	DPV	GQDs/IL/MWCNT/PANI/GCE	0.03 -12.0 μM	9 nM	Vegetable samples	(93)
Dopamine	DPV	Au-GQDs-Nafion/GCE	2 - 50 μM	0.84 μM.	Human urine sample	(94)
Tyrosinamide	EIS	N-acetyl-l-cysteine-capped Ag-In-S QDs/GCE	0.01 to 2.81 nM and 2.81–10.81	3.34 pM	Human serum sample	(95)
Bisphenol S	DPV	CQD/ AgNP /MIP/GCE	10 nM-0.05 mM	11.2 nM	Plastic products	(96)
Pimozide	DPV	NH2-fMWCNT/ decorated with and ZnONPs/ GQD/GCE	0.0625-120nM	0.0102 nM	Pharmaceutical samples Human serum sample	(97)
Uric acid	DC-AMP	CQD/ Fe3O4/GCE	0.01-0.145 μM	6 nM	Human urine sample	(98)
Diethylstilbestrol	LSV	GQD/SPCE	0.05 -7.5 μM	8.8 nM	Human urine sample Tap water	(99)
Paracetamol	DPV	PS-PNIPAm-PS / COOHfMWCNT-GQDs / GCE	0.1-7.0 μM 7.0-103.0 μM	66 nM	Human serum sample Pharmaceutical samples	(100)
Hydroquinone	DPV	CuO-His-GQD/GCE	0.001–40 μM	0.31 nM	Natural water samples	(101)
Dopamine	LSV	CQDs/GCE	0.19 – 11.81 μM	2.7 μM	-	(34)
Uric acid			0.21 – 13.39 μM	1.3 μM		
Amoxicillin	SWV	QDs-P6LC-PEDOT:PSS/GCE	0.90–69.0 μM	0.05 μM	Milk sample Human urine sample	(35)
Bisphenol S	DPV	hNiNS/GQDs/MIPs/GCE	0.1–50 μM	0.03 μM	Plastic samples	(102)
Epinephrine	SWV	GQD-CS/CPE	0.36–380.0 μM	0.0003 μM	Human serum sample	(103)
Arginine	DPV	fMWCNT/CdSe/HF-PGE	0.287–33670 μM	0.081 μM	Real samples	(104)
Alanine						
Methionine						
Cysteine						
Amino acids						
Riboflavin	DPV	N-CQD/SnO2/SPE	0.05–306 μM	8 nM	B complex tablet Riboflavin tablet Milk powder	(105)
Cisplatin	DPASV	GQDs-thio/npGCE	0.2-110 μM	0.09 μM	Human serum sample Human urine sample	(106)
Vitamin C	DPV	PPy-BPQDs-MIPs/PEDOTNRs/GCE	0.01–4 mM	0.0033 mM	Soft drink : Glucose Nicotinic acid Caffeic acid Folic acid	(107)
Calycosin	DPV	PAGD/GCE	11 μM -0.352 mM	9.8 μM	Astragali Radix	(108)
Dopamine	DPV	GQDs/GCE	0.4- 100 μM	0.05 μM	Real Sample	(109)
Hydroquinone Catechol	DPV	GQDs/GCE	0.5-100 μM	0.08 μM	River water samples	(32)
Dexamethasone	DPV	GNP/GCE	0.1–50 μM and 50–5000 μM	15 nM	Human serum sample	(110)
Amitriptyline	DPV	MagNPs/CQD/GCE	0.05–13.50 μM	0.0059 μM	Uric acid, Ascorbic acid, Dopamine, Estriol 17b-estradiol	(111)
Melatonin				0.0044 μM		
Tryptophan				0.0042 μM		
Ciprofloxacin	DPV	LDH/CdTe QD/CPE	25 nM- 12 μM	42 nM	Zn2+, Fe2+, Cu2+, Citric acid Ascorbic acid	(112)
Norepinephrine	SWAdASV	GQD/AuNP/GCE	0.5–7.5 μM	0.15 μM	Pharmaceutical samples Rat brain tissue	(113)
Dopamine	DPV	SnO2/N-GQD/PANI/GCE	0.5–200 μM	0.22 μM	L-ascorbic acid Uric acid solution	(114)
Hydrazine	CV	CdSe @ NiHCF NPs/electrode	1.6–1000 μM	0.5 μM	Tap water Seawater	(115)
Ascorbic acid	DPASV	GO/CdTe QDs/GCE	32.3–500.0 μM	6.1 μM	Fruit juice	(116)
Acetaminophen	DPV	GA@O-CQDs/GCE	0.001–10 μM	0.38 nM	Pharmaceutical samples	(68)
Carbendazim	DPV	ZnCdTe QD-rGO/CPE	99.8 nM -11.8 μM	91.6 nM	Orange juice	(117)
L- Tryptophan	DPV	NH2-GQDs/β-CD/GCE	1.0–30.0 μM	0.65 μM	10 mM Phosphate buffer (pH 7)	(118)
D-Tryptophan				0.12 μM		
L-cysteine	DPV	AgNPs/GQDs/GCE	0.2mM-10 μM	10 nM	-	(119)
Phenylethanolamine A	CV	MIP/C3N4NTs@GQDs/Ru@AuNPs/GCE	1 pM-1 nM	0.2 pM	Human urine sample	(120)
Uric acid	CV	UOx/GQDs/GCE	1–800 μM	0.3 μM	Human serum sample	(121)
Ascorbic acid	DPV	rGO/CdSeQD/GCE	0.39–1.0 mM	66 μM	Human urine sample	(122)
Dopamine			4.9-74 μM	0.11 μM		
Uric acid			9.0 μM –0.12mM	0.12 μM		
Estradiol	DPV	GQDs/ PSSA/GCE	0.001–6.0 μM	0.23 nM	Human serum sample	(123)
Progesterone				0.31 nM		
Alprazolam	DPV	Ag/N-GQD/Au electrode	56–156	56	Human serum sample	(124)
Diazepam			54–142	54		
Clonazepam			84-625	84		
Oxazepam			54–454	54		
Chlordiazepoxide			52–250	52		

## Conclusion

The field of electrochemistry and nanomaterials are areas in which researchers are increasingly interested in pharmaceutical and pharmaceutical analysis. In voltammetry, more sensitive and selective analyzes can be performed with the use of nanomaterials. Quantum dots are mostly used for enhancing electrochemical sensor performances. Carbon-based quantum dots and semiconductor quantum dots get much attention thanks to unique quantum properties and signal amplifying characteristics. Moreover, carbon quantum dots are known as zero-dimensional nanocarbon material and show unique electron-transfer abilities and an increment of large surface area and rich surface functional groups.

It is hoped that more attention will be paid to the development of modern electroanalysis with emphasis on simplicity and modification of electrodes for the quality of drug analysis. This review aims to discuss some examples of the use of electroanalytical applications in the analysis of drugs with quantum dots modified electrodes and to give detailed information about these applications. The pharmaceutically active compounds in the selected publications are reported in detail on the table in alphabetical order. The table presents the available information about the electrode type and modification agent, method, media, application sample, linear range, and detection limit. In this review, analytical applications of selected publications’ drugs using electrochemical methods are discussed. This review provides an overview of the analysis of aliquots with selected quantum modified electrodes using the voltammetry method.

## Future Prospects

The quantum dots-based electrochemical nanosensors are becoming quite a well-known sensor in recent years due to their outstanding features. The future perspective of electrochemical sensors in pharmaceutical and biomedical analysis. Over the last few years, electrochemical nanosensors incorporation of quantum dots such as carbon quantum dots, graphene quantum dots, and semiconductor quantum dots are widely utilized to fabricate sensing platforms exhibiting better redox properties. Aptamer and MIP-based biosensor is widely fabricated by modified the electrode surface with Quantum dots. Furthermore, fluorescent or colorimetric-based processes are being facilitated by the incorporation of quantum dots-based sensing for the rapid detection of pharmaceutical and biomedical analysis. Moreover, the fabrication of a miniaturized sensing platform has overcome the gap between detection in a diagnostic laboratory and point-of-care detection. The future objectives of quantum dots-based electrochemical nanosensors development should be designing on-spot measurements and commercialized them at minimum cost.
